# Diploid mint (*M. longifolia*) can produce spearmint type oil with a high yield potential

**DOI:** 10.1038/s41598-021-02835-6

**Published:** 2021-12-07

**Authors:** Nestor Kippes, Helen Tsai, Meric Lieberman, Darrin Culp, Brian McCormack, Rob G. Wilson, Eric Dowd, Luca Comai, Isabelle M. Henry

**Affiliations:** 1 Department of Plant Biology and Genome Center, University of California at Davis, UC Davis Genome Center, Davis, CA 95616 USA; 2 ANR, Intermountain Research and Extension Center, University of California Cooperative Extension, Tulelake, CA 96134 USA; 3 Ingredient Science, Mars Wrigley, 1132 W. Blackhawk St, Chicago, IL 60642 USA

**Keywords:** Plant breeding, Plant genetics, Secondary metabolism

## Abstract

Mint oil is a key source of natural flavors with wide industrial applications. Two unbalanced polyploid cultivars named Native (*Mentha Spicata* L) and Scotch (*M.* × *gracilis Sole)* are the main producers of spearmint type oil, which is characterized by high levels of the monoterpenes (−)-carvone and (−)-limonene. These cultivars have been the backbone of spearmint oil production for decades, while breeding and improvement remained largely unexplored, in part, due to sterility in cultivated lines. Here we show that sexual breeding at the diploid level can be leveraged to develop new varieties that produce spearmint type oil, along with the improvement of other important traits. Using field trials and GC-FID oil analysis we characterized plant materials from a public germplasm repository and identified a diploid accession that exhibited 89.5% increase in oil yield, compared to the industry standard, and another that produces spearmint type oil. Spearmint-type oil was present at high frequency in a segregating F_2_ population (32/160) produced from these two accessions. Field-testing of ten of these F_2_ lines showed segregation for oil yield and confirmed the production of spearmint-type oil profiles. Two of these lines combined high yield and spearmint-type oil with acceptable analytic and sensory profiles. These results demonstrate that spearmint-type oil can be produced in a diploid background with high yield potential, providing a simpler genetic system for the development of improved spearmint varieties.

## Introduction

Mint oil is produced when steam distillation of mint hay releases the oil stored in the leaf glandular trichomes^1,2^. Historically, the industry has relied on a handful of mostly “heritage” cultivars to produce the two main types of oil flavors, peppermint and spearmint. The complex genetic makeup of the few available cultivated varieties, frequent sterility, disease susceptibility, and lack of sexual breeding platform all represent key challenges to secure the sustainability of mint farming.

Diploid mints (genus *Mentha*) include *M. suaveolens* and *M. longifolia*, two of the progenitors of cultivated polyploid mints. Diploid mints are generally fertile, while most of the commercial polyploid hybrids are not. This is linked to ancestral, consecutive, and interspecific hybridization and whole-genome duplication events leading to polyploidy^3^. Two independent hybridization events produced the commercially available polyploid cultivars, the first one between the diploids *M. longifolia* and *M. suaveolens* produced spearmint (*M. spicata*). A second hybridization event between *M. spicata* and *M. aquatica* (octoploid) produced peppermint (*M. x piperita*)^4–6^. As a result of these consecutive interspecific events, cultivated mints carry complex polyploid genomes and vary wildly in chromosome number and composition^3,7,8^. Mint selection and cultivation has traditionally been based on sensory, and later analytical screens of the mint oil produced by each variety, with little or no information about their genomic composition. For example, Black Mitcham, currently the most popular variety for peppermint-oil production in the US, is a sterile hexaploid plant whose cultivation dates back to England in the eighteenth century and it is still the main producer of US peppermint oil^9,10^. The oil that Black Mitcham produces is considered the standard peppermint oil. “Native” and “Scotch” are two other polyploid cultivated varieties that produce spearmint type oil but with slightly different flavor profiles (*M. spicata L* and *M.* × *gracilis Sole* respectively). Both peppermint and spearmint oil yields have increased steadily between 1960 and 2000, mostly thanks to better agricultural practices, but also a regional shift from Western Oregon to south-central Washington and Idaho. Since then, mint oil yields have remained mostly stagnant, with incremental improvements due to better agronomic practices but no contribution from genetic improvement^9^. The combination of stagnant yields with the lack of expansion of mint acreage in the US in the last decades presents a challenge for the long-term supply of high-quality mint oil, and a great opportunity to produce transformative improvements in this industry such as more efficient farming and more flavor choices for consumers.

The major components of mint oil that provide flavor and aroma are isoprenoids, mostly volatile monoterpenes and sesquiterpenes^11^. Peppermint and spearmint oils are complex mixes of many different monoterpenes and sesquiterpenes, present at different concentrations^12^. Peppermint oil is characterized by high levels of (−)-menthol, a compound that provides a cooling sensation by binding the mammalian epithelial thermoreceptor TRPM8^13^. Spearmint oil contains high levels of (−)-carvone and (−)-limonene^14,15^. Carvone isoforms are a critical flavor component of many herbs such as caraway (Carum carvi) or dill (Anethum graveolens)^15,16^, and (−)-limonene has a piny, turpentine-like odor, unlike the D-isomer, (+)-limonene, which has citric aroma characteristic of many important fruits such as orange, lemons, or grapefruits^17^. The enzymatic steps leading to mint oil production take place in specialized structures found as extrusions on plant surfaces called glandular trichomes^14^. In these specialized cells, the acyclic precursors, geranyl pyrophosphate (GPP) and farnesyl pyrophosphate (FPP) are converted into an array of mono- and sesqui-terpenes, respectively, via pathways that have been well described for the most abundant compounds^14,15,18^. Yet, the effect of natural allelic diversity in structural and regulatory genes of these pathways is largely unknown, presenting a great opportunity for the discovery of new genetic variants to modulate the relative abundances of the individual flavor components.

Here, we characterize the oil of the mint diploid ancestors and present data that suggest that acceptable spearmint oil can be produced by leveraging the diploid species available in a public germplasm repository via new varieties produced through sexual reproduction. The discovery of a suitable spearmint flavor profile, and of high oil yield potential suggests a unique potential for mint improvement through diploid genetics.

## Results

### Diploids accessions as source of natural genetic variation for important mint traits

**Figure 1:**
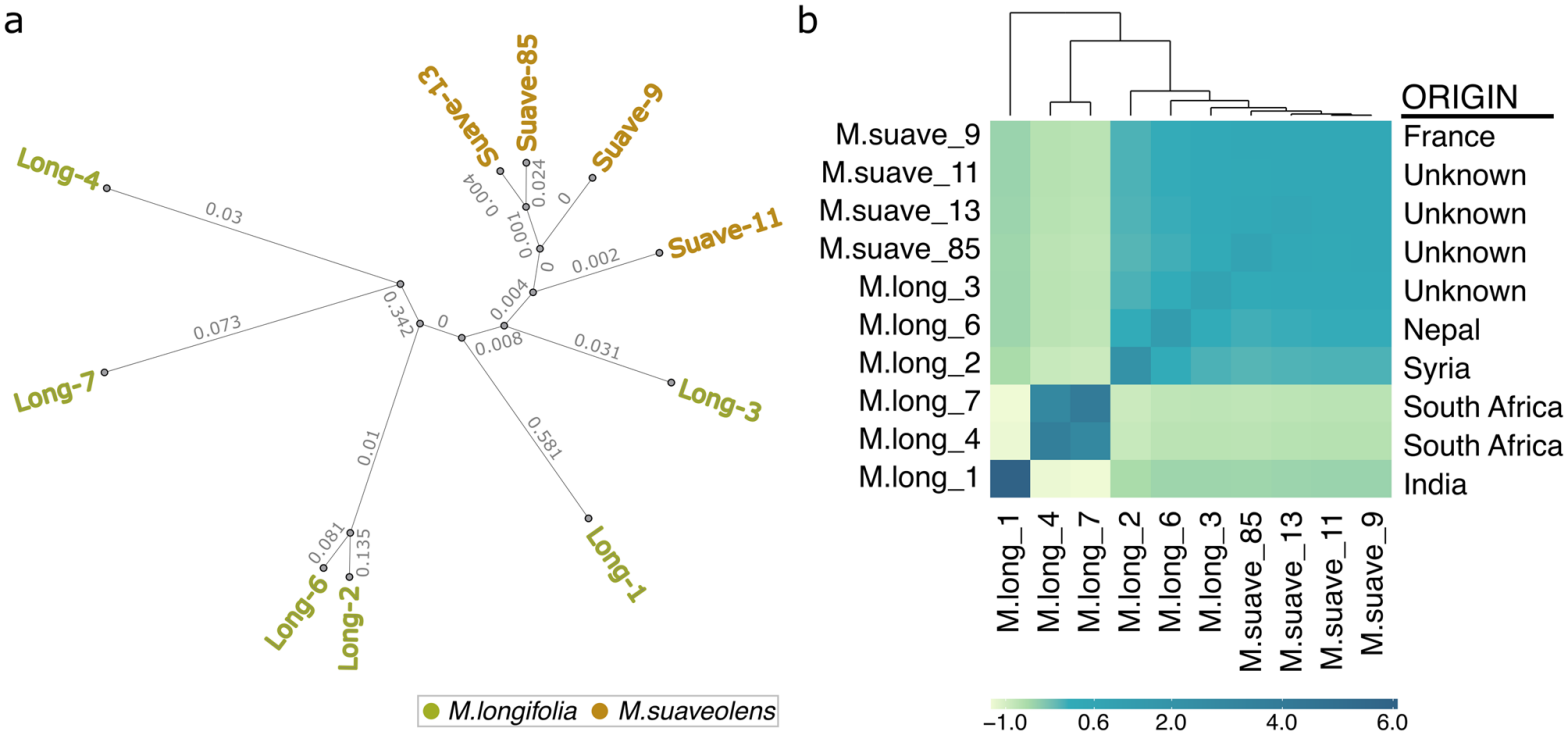
Population structure analysis of the accessions used in this study. (**a**) Phylogenetic tree based on SNP markers (Neighbor joining). Colors in name labels indicate species classification according to the taxonomy information in passport data. (**b**) Representation of kinship analysis calculated with 26,360 SNPs markers. Dark colors represent high similarity. M.long_4 and M.long_7 were found to be closely related. A detail description of these accessions is available in Table S1.

In order to identify natural genetic diversity with feasible practical applications within *Mentha*, we explored lines publicly accessible at the National Clonal Germplasm Repository (USDA ARS Corvallis, OR). We started by sequencing lines from the diploid progenitors of polyploid mints *M. suaveolens* and *M. longifolia* to establish genetic relationships. A set of 26,360 SNPs markers was obtained by whole genome sequencing of four *M. suaveolens* and six *M. longifolia* accessions. A phylogenetic analysis along with a kinship exploration showed that these are highly diverse. Three clusters were formed in both analyses, the first one including accessions from both species (Fig. 1), the second one including two *M. longifolia* accessions (M.long_4 and M.long_7), and the third including only one *M. longifolia* accession (M.long_1) that was found to be the most divergent within this set (Fig. 1). Collection location was obtained according to the passport data in the Germplasm Resource Information Network database (GRIN, USDA). Overall, collection site information was consistent with genetic diversity. M.long_1, the most divergent accession, was collected in India. M.long_2 (Syria), M.long_3 (unknown) and M.long_6 (Nepal) clustered with *M. suaveolens* accessions of unknown locations, except for one with a European origin (M.suave_9, France). Finally, M.long_4 and M.long_7 clustered together in a different group from the rest of the accessions, and both were collected in South Africa.

**Figure 2:**
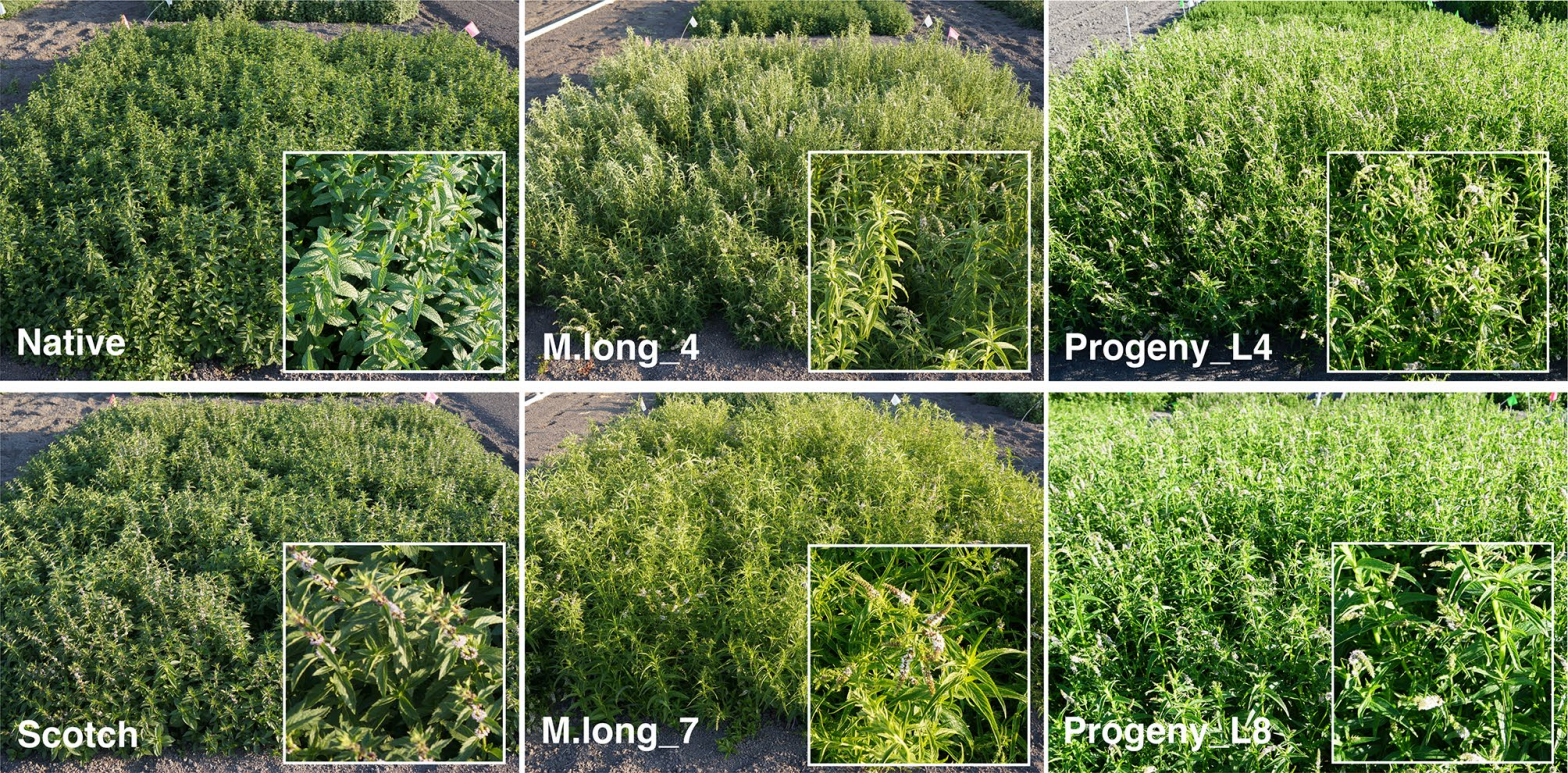
Field trial for mint oil yield. Representative pictures of the plots at maturity. Scotch and Native represent the industry controls for Spearmint type oil (polyploids), M.long_4 and M.long_7 are the parental *M. longifolia* accessions (diploids), and Progeny_L4 and Progeny_L7 correspond to two plants from the diploid F_2_ population.

A germplasm collection including some of these accessions has been characterized during the last decades in detail, in order to establish a core collection representative of mint genetic diversity^10,19^, but data on oil performance and quality under field conditions is lacking. We selected lines to explore field performance, including the accessions detailed above from the diploid species *M. longifolia* and *M. suaveolens*, plus a tetraploid *M. spicata* accession, as well as the two industry controls for spearmint type oil, Native and Scotch, also polyploids (see “Methods” for details). Using replicated plots, we produced oil from distillation of mint hay at the Intermountain Research and Extension Center at Tulelake California in 2018 (season 1) (Fig. 2).

We characterized the materials in terms of plant height, plot coverage, bloom at harvest, dry biomass and oil yield. All accessions presented good coverage of plots overall, ranging between 87–97 percent of the plot completely covered, with the lower values found for the *M. spicata* (M._spicata_88) and the Native spearmint control. Biomass ranged between 3.47 ± 0.22 – 6.89 ± 1.16 ton/ha where *M. suaveolens* accessions were high producers of dry biomass compared to other accessions (Table S2). The controls and *M. longifolia* accessions produced similar biomass and less than *M. suaveolens.* The controls showed a reduction of 29 or 41% of biomass (Scotch and Native respectively) compared to the highest biomass producer (M.suave_85, Table S1). In terms of plant height, the controls were amongst the shortest plants in the trial, with plant heights between 43–44 cm (Native and Scotch), where M.spicata_88 and M.long_3 were the shortest (41.83 ± 1 and 42.75 ± 2.55 cm respectively) and M.long_7 the tallest (78.42 ± 1.77 cm). A ten percent bloom is considered optimal for the timing of harvest and we found extensive differences in timing of maturity within these accessions. The controls Native and Scotch were at 1 and 14.25 bloom percentages respectively at the time of harvest, whereas all diploid species were much more advanced, with values ranging from 19.5 to 100 percent, with the exception of M.suave_85 that was at 6.5 percent bloom. Even though the appropriate timing of harvesting (~ 10% bloom) was optimized historically for commercial lines, and we do not necessarily know the effect of bloom percentage on non-commercial lines. Oil yield assessment could be optimized by identifying appropriate harvesting times for each accession.

**Figure 3:**
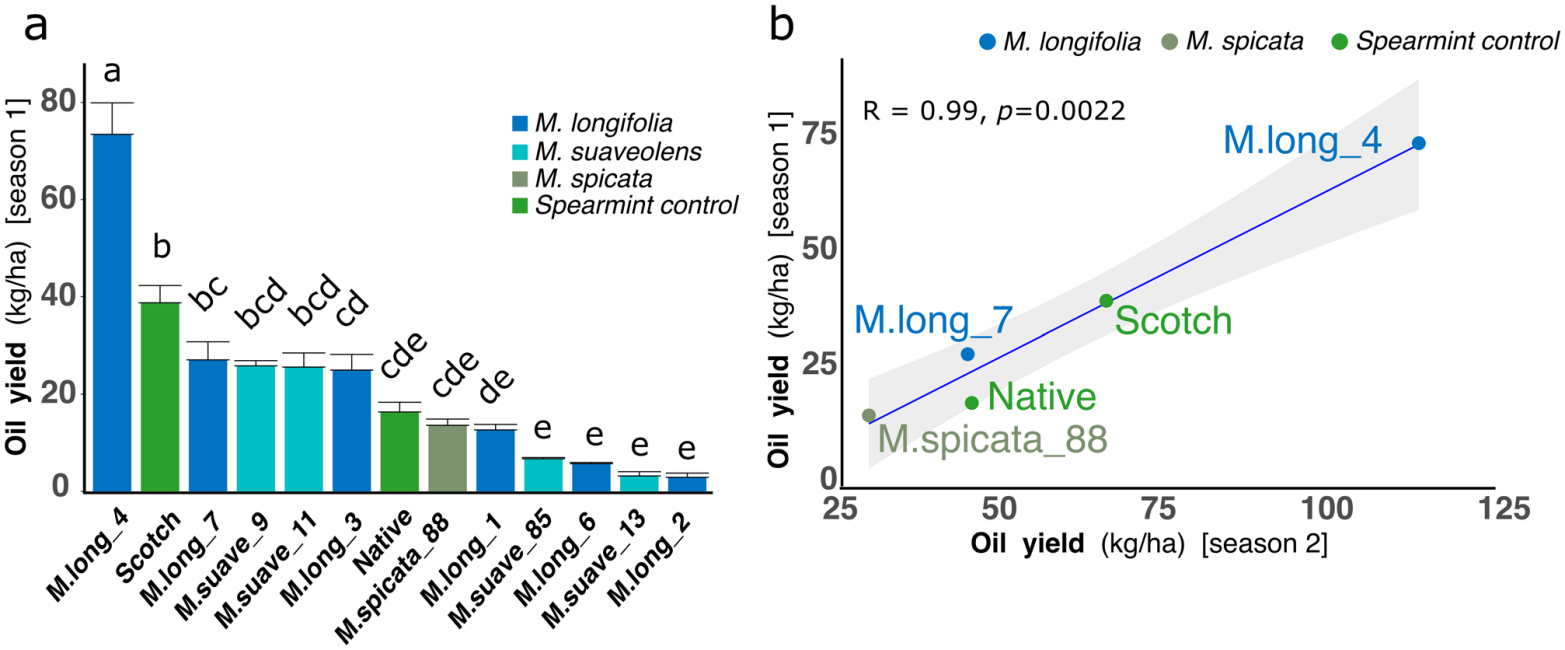
Mint oil yield variation under field conditions. (**a**) Mean oil yield values from four biological replicates of USDA accessions. Bars represent standard errors and different letters indicate significantly different means (Tukey, *p* < 0.05). (**b**) Relationships between oil yield in season 1 (2018) and season 2 (2019) for 5 cultivars characterized both years. Each data point represents the mean of 3 (2019) or 4 (2018) biological replicates (plots). Native and Scotch are the industry controls for spearmint flavor, *M.spicata*_88 is a polyploid accession that produces low-quality spearmint oil.

Oil yield exhibited a wide variation, with values ranging from 2.89 to 73.43 kg/ha. Overall, most lines yielded less, or at levels similar to the controls, with the exception of one *M. longifolia* accession that was the top yielder (M.long_4), representing an increase of 89.5% compared to the spearmint control Scotch (Fig. 3a).

Most of the traits showed small or no significant correlation to each other. The only significant correlation found was a positive correlation between dry biomass and plot coverage (*p* < 0.0001, Fig. S1). GC-FID analysis showed extensive variation for oil composition. From 78 identified compounds in total, only 10 compounds were found in all accessions, including 1,8-cineole and (−)-limonene, and most of them at low abundances (Table S4, Fig. S2). Four out of the six *M. longifolia* accessions studied produced isoforms of piperitenone oxide as main component of the oil (35–59%), and the remaining two produced very distinct profiles containing pulegone (M.long_4) or (−)-carvone (M.long_7) as main components. Between the four *M. suaveolens* accessions studied, two produced (−)-carvone as main component at ~ 40% levels (M.suave_9 and M.suave_11), and two produced piperitenone oxide as main component at ~ 32% level (M.suave_13 and M.suave_85) in agreement with a previous report of the most abundant compounds on these *M. suaveolens* accessions^25^. The complete data on each of these accessions is available in Table S4.

**Figure 4:**
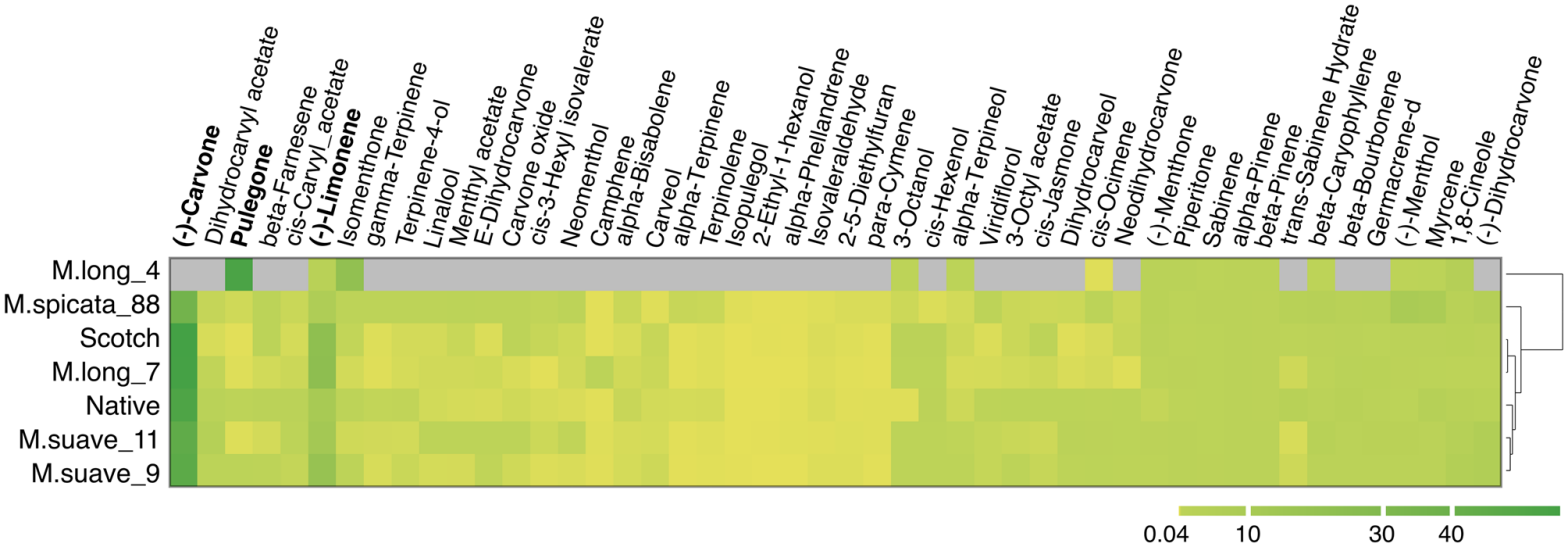
Oil composition under field conditions. GC-FID analysis of oil samples obtained under field conditions. Native and Scotch are the industry controls for spearmint flavor (polyploids), M. long corresponds to different *M. longifolia* accessions (diploid) and M.suave to *M. suaveolens* accessions (diploid), *M.spicata*_88 is an *M. spicata* accession (tetraploid) that produces low-quality spearmint oil. The matrix displays mean relative abundances (% relative to the peak area) from four biological replicates. Gray color indicates that a compound was not detected. Data from additional samples are presented in Table S4 and a hierarchical clustering of analytic composition is presented in Fig. S2.

To further characterize the observed variation in oil composition, we selected the accessions that produced oil with (−)-carvone as the most abundant compound, and the *M. longifolia* accession with the highest oil yield, and clustered the samples based on the relative abundances of oil components (Fig. 4). Two *M. suaveolens* accessions clustered with one of the controls (Native), and one *M. longifolia* (M.long_7) presented the closest similarity to the spearmint oil shown by the Scotch control (Fig. 4 and Fig. S2). These profiles were characterized by high levels of (−)-carvone and (−)-limonene (Fig. 4). M.long_7 produced oil with (−)-carvone and (−)-limonene levels of 55.54% and 23.69%, respectively, whereas the Scotch control produced oil with (−)-carvone and (−)-limonene levels of 55.81% and 22.98%, respectively (Fig. 4, Table S4).

The accession that exhibited the highest oil yield (M.long_4) produced an oil with a profile that was very different from spearmint, with no detectable levels of (−)-carvone, and in which the main components were pulegone (53.15%), a compound with undesirable sensory characteristics, and isomenthone at 21.48% as the second most abundant compound (Fig. 4, Table S4).

### A segregating diploid population for spearmint type oil

The discovery of individual accessions with either high yield, or good analytic profiles for spearmint oil type in a diploid background presented a great opportunity to explore the possibility of combining these characteristics by sexual reproduction. Specifically, these two accessions (M_long_4 and M_long_7) are diploid, fertile, genetically very close (Fig. 1), and present similar phenotypic characteristics. They were crossed to produce F_1_ progeny using M.long_4 as female. The hybrid nature of each F_1_ progeny was confirmed by Sanger sequencing of two PCR markers (Table S6). Next, three F_1_ plants were selfed to produce 160 F_2_ seeds in total. F_2_s were grown to maturity and a subset was selected based on aroma, focusing on lines exhibiting similarities with the Scotch spearmint control, and low pulegone levels. Indeed, pulegone, the main component in one of the parental lines (M.long_4), was used as a marker for undesired characteristics since it produces an unpleasant aroma and is easy to identify at greenhouse level. Based on this criteria, ten lines were identified for field testing and planted along with the parental controls using randomized complete blocks experiment designs. During this second year of field testing, the main phenotypic differences previously observed between the two *M. longifolia* parental lines were maintained: the two lines had contrasting plant heights (64.17 cm and 84 cm for M.long_4 and M.long_7, respectively), and oil yields (115.25 ± 12.84 and 44.58 ± 2.95 kg/ha for M.long_4 and M.long_7, respectively). Oil yields in the control lines, as well as the parental accessions in both seasons were well correlated (Fig. 3b, R = 0.99, *p* = 0.0022). Both lines also produced similar amounts of dry biomass (4.77 ± 0.53 and 4.9 ± 0.28 ton/ha for M.long_4 and M.long_7, respectively) while reaching similar levels of maturity at harvest (40.0% and 37.5%, respectively, Table S3).

**Figure 5:**
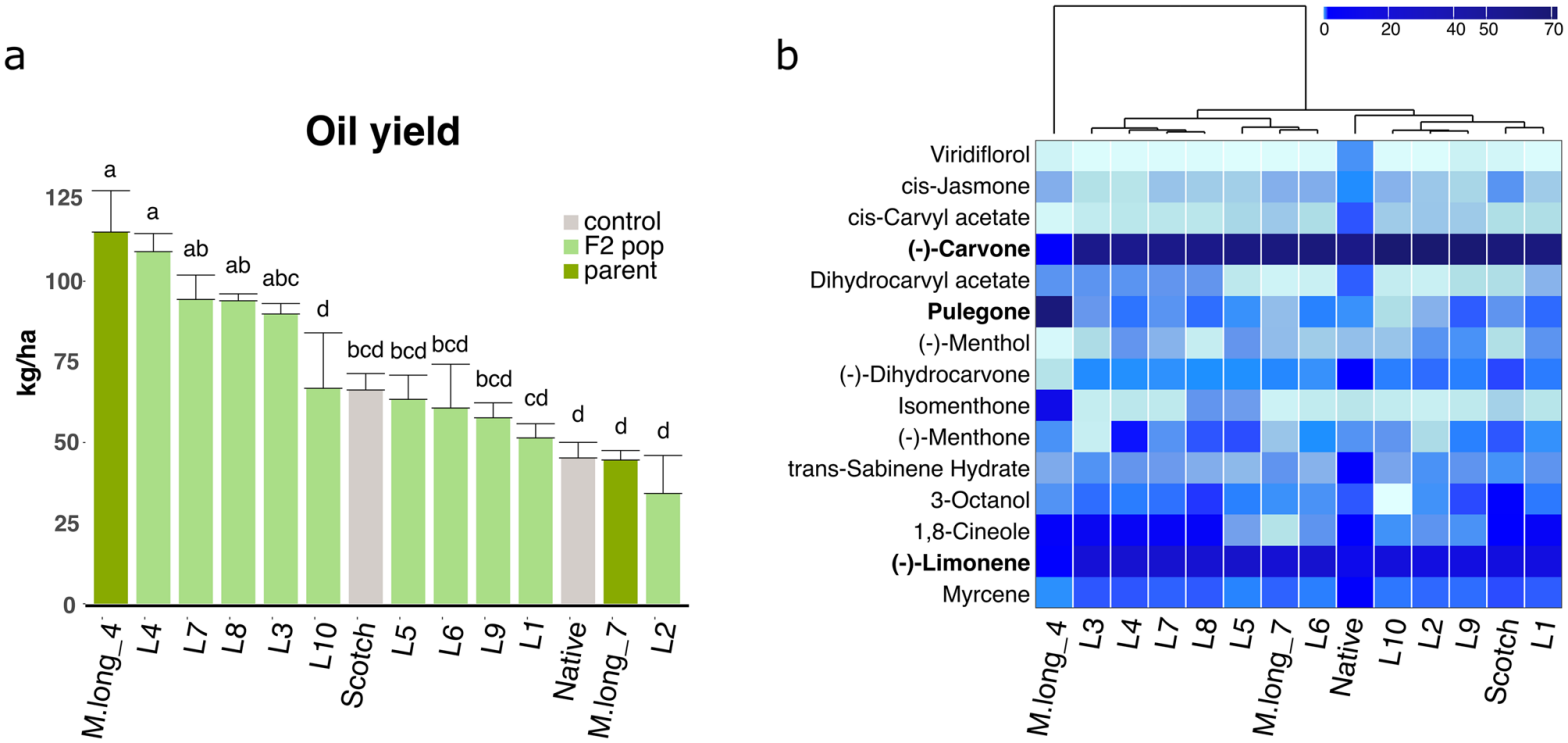
Characterization of diploid progenies under field conditions. M.long_4 and M.long_7 are the parental accessions, Native and Scotch are the industry controls for spearmint flavor. All other samples (L1–L10) represent F2 progenies from an M.long_4 × M.long_7 cross. (**a**) Oil yields in F2s, control lines and parental lines. Bar heights represent the mean of three biological replicates. Standard errors are indicated. Lines L3, L4, L7 and L8 all exhibited statistically significant increases in oil production, compared to the industry control (Scotch spearmint) (*p* < 0.05). (**b**) Heatmap of oil composition profiles based on GC-FID analysis of oil samples. The matrix displays mean relative abundances (% relative to the peak area) from three biological replicates. The parents of the F_2_ progenies have distinct analytic profiles: the main component of M.long_4 oil is pulegone while, in M.long_7 oil, the most abundant compound is carvone with very low pulegone levels.

Overall, the selected F_2_s exhibited segregating values for the measured traits, including plant height (50.56 cm–86.78 cm), oil yield (34.21 ± 11.7–109.18 ± 12.8 kg/ha), as well as for dry biomass (2.54 ± 0.98 ton/acre–6.7 ± 0.04 ton/ha). Four F_2_ lines produced 35.5 to 64.7% higher yields (89.86 to 109.18 kg/ha, Fig. 5a) than the Scotch control (66.29 kg/ha). These lines also produced more biomass than the control (41 to 67%). We found a positive correlation (R = 0.53, *p* = 0.049) between oil yield and dry biomass in all the lines tested (Fig. S3), and a higher correlation in the progenies (R = 0.84, *p* = 0.0026).

GC-FID analysis of the oil obtained confirmed that the parental accessions exhibited contrasting analytic profiles, with pulegone as the most abundant compound in the oil of M. long_4, and (−)-carvone in M. long_7, in agreement with the analytic profiles of these accessions in the previous growing season (Fig. 4). M. long_4 exhibited the most distinct oil profile of the whole dataset (Fig. 5b and Table S5). The F_2_ lines clustered into two groups, one including the progenies L3, L4, L7, L8, L5, L6 and M.long_7, and the second one containing the progenies L1, L2, L9, L10 and the spearmint controls. The most abundant compounds in both groups were (−)-carvone, (−)-limonene and 1,8-cineole, all at similar levels in both groups. (−)-Carvone levels were very low in M.long_4 (2.63%) compared to M.long_7 (63.9%), and 66.4% and 59.8% in the controls (Scotch and Native respectively, Fig. 5b, Table S5). (−)-Carvone levels in the progenies were high, ranging between 56.5% (L4)–71.6% (L2). A very similar tendency was observed for (−)-limonene, for which levels were 1.92% in M.long_4, 21.8% in M.long_7, and 16.8% and 10.7% in the controls (Scotch and Native respectively, Fig. 5b, Table S5). (−)-Limonene levels in the progenies ranged between 15.84% (L2) and 26.31% (L5). The third most abundant compound was 1,8-Cineole, with contrasting levels in the parents (3.5% in M.long_4 and 0.06% in M.long_7), and levels between 0.14% (L5) to 7.53% (L3) in the progenies, whereas the controls showed levels of 1.54% (Scotch) and 2.27% (Native).

Pulegone is another compound with contrasting abundances levels in the parents. As observed in the previous year, it was very high in M.long_4 and low in M.long_7 (65.75% and 0.10% respectively). The progenies all presented very low levels of pulegone (0.07–0.89%), indicating that the sensory selection at greenhouse levels was sufficient to identify and avoid high pulegone plants. Other less abundant compounds in the parental lines such as l-menthol, isomenthone, l-menthone, cis-jasmone and viridiflorol, all remained at low levels in the progenies. The high yielding lines L4 and L8, with analytic profiles in line with the spearmint controls, composed by high levels of (−)-carvone, (−)-limonene and 1,8-cineole and low levels of pulegone were classified as acceptable spearmint types by a sensory analysis (see “Methods”) and no unacceptable sensory notes were detected.

### Characterization of oil quality and chemotypes relationships

The flavor and aroma characteristics of mint oil arise from a complex mix of monoterpenes and sesquiterpenes. Monoterpenes, the most abundant terpenoids in mint oil, are produced by conversion of the universal monoterpene precursor geranyl diphosphate (GPP)^14,15,18^. Most of the enzymes characterized in detail belong to peppermint, and (−)-menthol synthesis specifically has been the focus of the mint biochemical research and engineering^14,20^. Here we characterized the relationship between the abundance of different compounds. To help interpret these results, we assume that spearmint (*M. spicata*) and peppermint (*M x piperita*) share similar enzymatic pathways. Phylogenetic relationships^5,21^ and the identification of many of these enzymes in *M. spicata* support these assumptions^2,20^.

**Figure 6:**
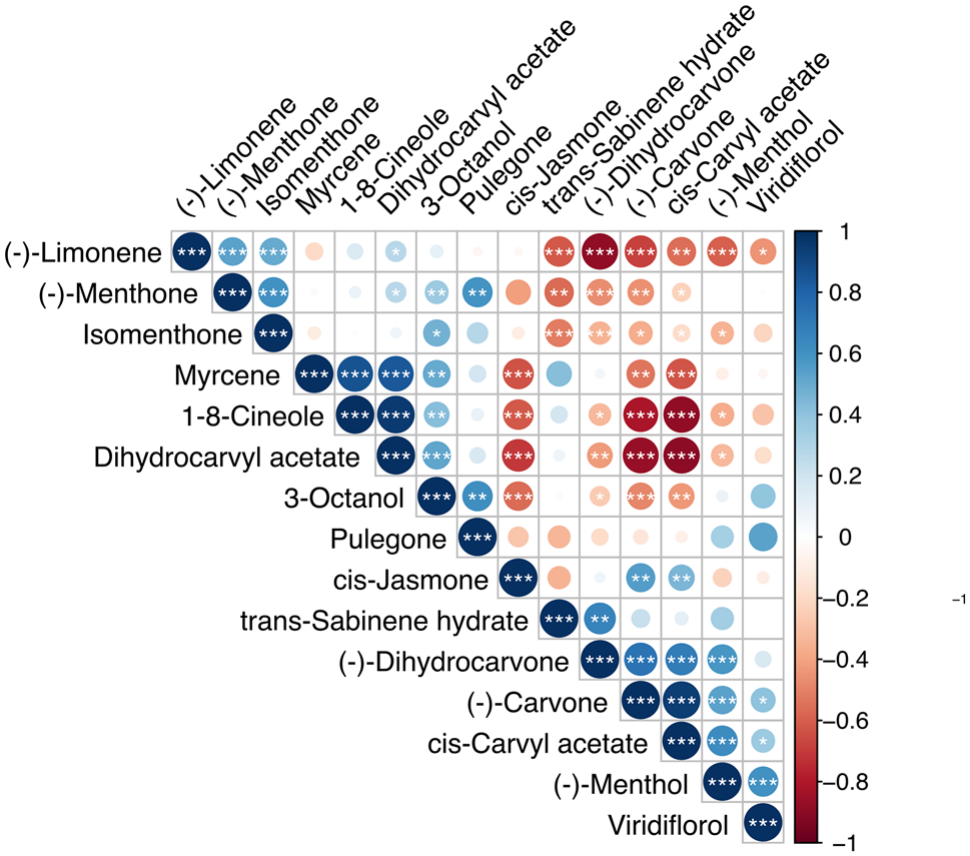
Correlation coefficients between the different compounds identified in the oils of F_2_ plants. We found both negative and positive correlations between different compounds identified. Size of the circle and color intensity are proportional to the correlation coefficients. Significant correlations are indicated by asterisks: *p*-value < 0.001 (***), *p*-value < 0.05 (**) and *p*-value < 0.01 (*).

1,8-Cineole and (−)-carvone were found to be negatively correlated in our field-tested progenies (Fig. 6), which is consistent with the fact that these two compounds compete for the same precursor (GPP). (−)-Carvone is produced by three sequential modifications of GPP, that start with the production of (−)-limonene^22^. Further modifications of (−)-limonene produce (−)-dihydrocarvone and cis-carvyl acetate, also found to be negatively colleraled with 1,8-cineole, indicating that metabolic branches that produce (−)-carvone or 1,8-cineole in *M. longifolia* present similarities with the model characterized in peppermint (*M x piperita*) and spearmint (*M. spicata*).

(−)-Limonene was found to be negatively correlated with cis-carvyl acetate and (−)-carvone (Fig. 6). These last two compounds are produced by different modifications of (−)-limonene, suggesting that the negative correlation is due to differential enzymatic preferences to produce (−)-limonene modifications.

An alternative branch of (−)-limonene modifications leads to the production of pulegone through four enzymatic steps. Two additional modifications eventually lead to (−)-menthol^20^, as one of the best characterized metabolic pathways in plants^14,20^. Consistently. In our analysis, (−)-menthol showed a clear negative correlation with (−)-limonene, and a small positive correlation with pulegone (an intermediary step in menthol production) and isomenthone, an alternative modification of pulegone (Fig. 6).

Myrcene, a compound with fruity, earthy and musky notes, is produced as a side product of (−)-limonene synthase catalysis over the precursor GPP^22^. Here we found positive correlations between myrcene and 1,8-cineole and dihydrocarvil acetate, and negative correlations between myrcene and (−)-carvone and cis-carvyl acetate (Fig. 6). In this case, the correlations are more difficult to interpret, suggesting that there may be additional catalytic steps between these compounds that are missing in the current model of the mint pathway. 1,8-cineole and (−)-limonene are described as alternative branches that compete for the GPP precursor^11^, but here we found a positive correlation with 1,8-cineole and a negative but small correlation with (−)-limonene.

Finally, (−)-carvone is produced by modifications of (−)-limonene. Dihydrocarvyl acetate could share the same precursor but this catalytic pathway is not completely described in mint^20,22^. Here, these compounds exhibited one of the most negative correlations found in the whole dataset (Fig. 6). The high levels of (−)-carvone and low levels of dihydrocarvyl acetate observed in the population suggest competition for the same precursor that favors (−)-carvone production.

## Discussion

### Natural genetic variation in key traits as resource for mint improvement

In the past decades, there have been great contributions to the characterization of the valuable materials including progenitors of polyploid mints such as the diploids *M. suaveolens* and *M. longifolia* and the octoploid *M. aquatica*. Taxonomic descriptions^5,23^, cytologic studies^24,25^, oil profiles^10,19^, disease resistance information, and morphological classifications^25^ are all foundational data leading to the development of breeding and improvement resources, and highlight the importance of plant conservation efforts for long term crop sustainability. In our study, we provide a detailed characterization of the genetic variation available for traits that are not commonly explored in plant conservation studies, such as oil yield, biomass production and oil quality under standardized field conditions with distillation at pilot plant scale. *M. longifolia* and *M. suaveolens* accessions, diploid ancestors of the polyploid *M. spicata,* showed diverse genetic makeup, and extensive variation for oil yield and quality. Our analysis of genetic diversity indicates that *M. longifolia* is a highly diverse subspecies. While all accessions of *M. suaveolens* grouped together in our kinship analysis, *M. longifolia* formed three distinct groups (Fig. 1b). The parental accessions used for the development of the diploid population presented a close genetic relationship in concordance with their geographical origin and formed a distinct group from other *M. longifolia* accessions. A previous analysis of *M. longifolia* phylogeny with SSR markers presented a similar result^19^. Clustering of the samples based on genetic similarity and oil composition produced very different results. The *M. suaveolens* accessions tended to group together in our genetic analysis (Fig. 1) while producing oils of substantially different compositions. Specifically, based on oil composition profiles, two of the *M. suaveloens* accessions clustered with the Scotch control, and two with other *M. longifolia* accessions (Fig. S2, Table S4). Similarly, the two *M. longifolia* lines used as parents in this study were genetically very similar (Fig. 1), but produced very distinct oil profiles (Fig. S2, Table S4). Together, this data suggests that genetic and analytical oil analyses are both needed to assess diversity and fully understand metabolic complexity.

Overall oil yields in the USDA accessions were below the Scotch spearmint control with some lines yielding ten times less than the control. But one line was exceptional, showing an 89% increase in oil yield compared to the industry control (Fig. 3a). These results indicate that high oil yield is not very frequent and highlights the importance of identifying the best parental accessions for future breeding efforts. This also demonstrates the need for direct measurement of oil yield: some lines that produced very low oil were high biomass producers, with up to 42.2% increase in biomass compared to the controls (M.suave_85,Table S2). In terms of oil yield or biomass production our analysis is limited by a single cut date for all accessions. Further studies of harvesting date optimization could maximize these traits in the materials studied.

At the molecular level, little information is available in terms of the limitations and possible exploitation of genes and alleles to increase mint oil production. One recent advance showed that, in some plant species, two enzyme families (isopentenyl phosphate kinases (IPK) and members of the Nudix hydrolase family) can modulate the carbon flux towards monoterpene production by interconverting diphosphate terpene precursors and their corresponding monophosphate^26^. Further experimentation on these topics could be highly beneficial to understand how oil yield production is modulated. So far, these enzymes have not been characterized in mint yet, but they offer a potential new approach to increase mint oil production. We expect that the information presented here along with the number of genetic markers produced in our study will help investigate these hypotheses.

The analytical composition of the oil produced under field conditions also showed extensive variation. If we analyze the main constituent in the oil, *M. longifolia* accessions produced variations of piperitone oxide, and the two lines showed pulegone (M.long_4) or (−)-carvone (M.long_7) as the most abundant metabolites. *M. suavelones* showed (−)-carvone or piperitone oxide as the primary compounds. These data support the hypothesis of hybridization between *M. longifolia* and *M. suavelones* to produce spearmint oil (*M. spicata*), with (−)-carvone as its main constituent^4,5^. Nevertheless, the analysis of the most abundant compound is an oversimplification of the flavor and aroma characteristic of mint oil. We were able to detect substantial differences between all the oils produced: from a total of 78 compounds identified, 11.5% were found in only one accession, 53.8% of compounds detected in six or less accessions, and only 12.8% of all compounds were identified in all accessions. Variation in the levels of the compounds characterized were also evident (Table S4). In our analysis, we were able to cluster one *M. longifolia* accession and two *M. suaveolens* with the spearmint controls (Fig. 4), showing that the selection of the spearmint flavor in the cultivated lines was not only guided by the main component of the oil, but rather by a complex interaction of many different compounds at different concentrations. The clustering of the analytic profiles of two *M. suaveolens* with the Native spearmint control, and one *M. longifolia* with the Scotch control suggests that those may have retained differential contributions from the diploid ancestors. Further detailed genetic studies to understand the different parental contributions to these cultivated polyploids could shed light into the nature of the hybridization processes that lead to optimal flavor characteristics.

### Diploid mint presents an opportunity for the development of new spearmint varieties

The versatility of mint to be clonally propagated has facilitated the selection of lines based on aromatic characteristics, along with a good field performance, but it has incorporated infertility and meiotic instability in the form of complex hybrids, impeding further combination of desired characteristics by sexual reproduction. The genetic complexity of the main cultivated spearmint lines present challenges for the establishment of a breeding scheme. Spearmint-type oil is produced mainly by two polyploid varieties, Native (*M. spicata*). and Scotch (*M.* × *gracilis Sole*). Native is a sterile triploid hybrid and Scotch is heptaploid (2n = 7x = 84)^19,25^. Public and private institutions have attempted to improve the historical peppermint and spearmint varieties during the last decades without much success in the form of new cultivars that can completely replace the historic ones. Mutation breeding was used in the 70’s as a tool to improve peppermint (another sterile complex hybrid) in terms of *Verticillium* resistance, the most devastating mint disease. These efforts met mixed results with the development of cultivars with increasing resistance but not to a level that could drive wide adoption^27^. The same strategy was used in spearmint (both Native and Scotch) to increase resistance to mint rust (*Puccinia menthae*) and *Verticillium* wilt with similar results^28,29^. Another approach to mint improvement entailed the combination of favorable characteristics, via sexual reproduction, to produce spearmint types using plants with different ploidy. For example, *M. arvensis* cv Kalka (2n = 8x = 96) and *M. spicata* cv Neera (2n = 2x = 24) yielded a pentaploid hybrid (2n = 5x = 60)^30^. These irregularities are typically eliminated in sexually reproducing plants because they are meiotically unstable or via competition among progeny, but can accumulate unhindered and be maintained in vegetatively propagated species such as mint^31^. These examples of atypical breeding strategies can potentially succeed in the production of an improved cultivars, but they face serious challenges when it comes to the complexity of producing plants exhibiting good flavor and good agronomic performance in a single step. More importantly, these lack the advantages of the establishment of a sexual breeding cycle, that can produce improvement in incremental and successive steps over long periods of time.

The lines produced in this study, by combination of two fertile diploid plants, segregated for oil yield, with values ranging from 34 to 109 kg/ha (Fig. 5a). All of these lines presented a high (−)-carvone/(−)-limonene and low pulegone levels, demonstrating that the increase in oil production is not linked to the high abundance of pulegone, or low levels of (−)-carvone/(−)-limonene, as in the parental high yielding parent (Fig. 5b). Four out of the ten plants selected by the sensory test at greenhouse level, presented an increase in oil production compared to the Scotch spearmint control, with increases of up to 64.7% (Table S3). Using the analytic information from these lines we identified strong correlations between the compounds present in the oil. Some of the most abundant compounds in spearmint oil, and key components of the aroma, such as (−)-carvone and (−)-limonene, showed the strongest correlations in the segregating progenies. Even though these plants were initially selected based on their aroma at the greenhouse level, before being tested under field conditions, these correlations found in the field are evidence of the interconnections of the synthesis of these compounds. Other less abundant compounds, but also important for spearmint flavor and aroma, such as myrcene, 1,8-cineole and (−)-menthol showed interesting correlations. For example, (−)-menthol presented a negative correlation with (−)-limonene which is used as a precursor in the (−)-menthol pathway, and positive with pulegone (2 enzymatic steps before menthol is produced) and isomenthone (and alternative branch of pulegone modifications). These data, mined from a relatively small number of plants, show that genetic variation for alleles of key aromatic components are tunable via selection of segregating material and opens the door to further opportunities of spearmint flavor improvement.

The data we present here indicate that sexual breeding for spearmint oil in a diploid and fertile background is feasible. Spearmint flavor, one of the most complex phenotypic characteristics, is already present in some *M. longifolia* accessions (Fig. 4), facilitating the improvement of other traits such a winter hardiness, yield stability and disease resistance, that could also be bred using a diploid genetic system where flavor and oil yield could be fixed around acceptable levels. For example, one of the parents used in our segregating population is completely resistant to *Verticillium* wilt^19^, making this an excellent scenario for deeper studies in unexplored areas such as the interactions between wilt resistance, oil yield and flavor characteristics.

### Natural alleles are key for future trait improvement

The increases in yield found in the germplasm collection suggest that the physiological limits of oil production in mint are far from being reached and present a great opportunity for improvement. The combination of spearmint flavor characteristics at levels similar to those found in the cultivated varieties, along with the increases in yield using a relatively small number of segregating individuals indicates that the control of these important traits is governed by a small number of loci (Fig. 5). Terpene biosynthesis has been studied in detail providing models for the metabolic pathways for functionalized monoterpenes^14,15,18^, the availability of high quality genomes, and the identification of relevant natural alleles are a key component for future studies that can incorporate tools, such as high throughput genotyping or genome editing, to improve our understanding of the biological process that govern the diversity of secondary metabolites. At the same time, mint embodies an ideal opportunity as a model species for other members of the Lamiaceae family, including aromatic plants and culinary herbs (basil, rosemary, thyme, sage, lavender, oregano, etc.), as well as other oil producing plant species with longer breeding cycles (citrus, eucalyptus, etc.), or plants species where aroma is a key component for fruit quality (e.g. peach) or end products (e. g. grapevine).

## Methods

### Plant materials

Plant materials including 6 M*. longifolia* accessions (M.long_1 PI557758, M.long_2 PI557770, M.long_3 PI557755, M.long_4, PI557767, M.long_6, PI557768, and M.long_7, PI557769), 4 M*. suaveolens* accessions (M.suave_S9, PI557638, M.suave_S11, PI557998, M.suave_S13 PI557891, and M.suave_S85 PI557898) and 3 M*. spicata* accessions (Native PI557885, Scotch PI557935, and M.spic_88 PI557802) were obtained from the National Clonal Germplasm Repository (USDA ARS Corvallis, OR). Plants were received as cuttings and maintained under greenhouse conditions. A population was developed by crossing M.long_4 (female) and M.long_7 (male) accessions. F_1_ seeds were cleaned with 0.17% bleach for 10 min, washed three times with sterile water, stratified in 0.5X agar media at 4 °C for 7 days and kept at room temperature for 7 days before being moved to soil under greenhouse conditions to produce seeds. F_2_ seeds were collected from three individual F_1_s, germinated and grown under greenhouse conditions to produce 160 plants. Ten F_2_ plants were selected based on their aroma under greenhouse conditions and grown until ready for clonal propagation and testing under field conditions.

### Genotyping and population structure analysis

All *M. suaveolens* and *M. longifolia* lines used in this study were sequenced using multiplexed barcoded libraries in a HiSeq4000 sequencer at the UC Davis Genome Center. Sequencing libraries were produced using the KAPA HyperPlus Library Kit (Kapa Biosystems, Wilmington, MA) following the manufacturer's instructions with a modification on the fragmentation step. We used a Covaris E220 sonicator at the factory settings, to replace the use of fragmentase enzyme, with a target size of 300 bp in 50 µl TE buffer. The fragmented DNA was purified using in-house magnetic beads^32^. After quality control checking (https://github.com/Comai-Lab/allprep) and checking for over amplified reads (https://github.com/Comai-Lab/overamp), the reads ranging from 18 to 37 million pair ended 150 bp reads per sample were mapped using BWA MEM (http://bio-bwa.sourceforge.net/) to the published *M. longifolia* genome^33^. The data was surveyed for the presence of single nucleotide polymorphism using a combination of samtools https://github.com/Comai-Lab/mpileup-tools) and the MAPS mutation and polymorphism detection pipeline (https://github.com/Comai-Lab/MAPS). Positions for which data was available for all libraries, and for which coverage was suitable (total coverage between 50 and 1,000 reads) were retained. Finally, only positions for which all lines presented adequate read coverage (10 or more) and homozygous base calls (> 95%), and for which at least one line presented a different base call as the others were retained, producing a total of 26,360 SNP positions. These were used to calculate a kinship matrix as implemented in TASSEL^34^. Additionally, a ten percent random subsample of markers (2,630) was used to build a phylogenetic tree using Neighbor-Joining method with the R packages ape (http://ape-package.ird.fr/), phangorn (https://github.com/KlausVigo/phangorn) and seqinr^35^.

### Field experiments

Field experiments were conducted at the University of California Intermountain Research and Extension Center field station at Tulelake, CA (41° 57′ 54.8ʺ N 121° 28′ 14.5ʺ W). Planting occurred in early May and harvesting in mid-September each year. The previous crops in these fields were spring and winter wheat in 2018 and 2019 respectively. Fields were hand planted using randomized complete blocks experiment designs with four replications/entry in 2018, and three replications/entry in 2019. In 2018, each plot included 42 plants in 150 sqft (13.93 m^2^), resulting in a plant density of 12,196 plants per acre (30,136 plants/ha). In 2019, 45 plants were planted on a slightly smaller plot of 120 sqft (10.76 m^2^), resulting in a plant density of 16,335 plants per acre (40,364 plants/ha). Spacing between plots was 6 ft in 2018 and reduced to 2 ft in 2019 to facilitate weed management. Plots were fertilized each year with nitrogen. In 2018, the field received 100 U/acre of granular urea (Urea 46-0-0, JR Simplot, USA) before planting and two applications of UAN 32% solution (UAN 32%, JR Simplot, USA) at 25U/acre during the season. In 2019, the field received 56U/acre of granular triple twelve NPK fertilizer (12-12-12 with 15% sulfur, JR Simplot, USA) and four applications of UAN 32% at 25U/acre during the season. Fields were watered using solid-set sprinklers. In 2018, 14 inches of water were applied in 13 applications, and 18 inches in 18 applications in 2019 (weather data available in Fig. S6). Weed control was performed manually as needed with the exception of the use of 44 oz of Roundup PowerMax prior to planting in 2018. The application of fungicides or insect control was not needed. Bloom percentage was calculated based on the proportion of open flowers at harvest. Plant height was calculated from the average of three measurements in each plot. The percentage of dry biomass was calculated by dividing the weight of a sample after complete desiccation in a 60 °C dryer by the weight of the sample at harvest. Total dry biomass was calculated using percentage of dry biomass multiplied by total weight of hay at harvest. Since plants reached maturity at different time points, and due to management and distillation constrains, we chose to perform a single harvest for all plants. In consequence, oil yield estimates are a reflection of oil yield potential as well as differences in maturity between lines, meaning that oil yields could be further optimized by studying the effect of different harvesting points on each line.

### Mint oil distillation

Oil was extracted using a pilot plant for steam distillation available at the University of California Intermountain Research and Extension Center field station at Tulelake, CA. This unit was built, based on the Mint Industry Research Council (MIRC, www.usmintindustry.com) standards and design, in 2004. Harvested material was left to dry in the field for two days before distillation (flipped over after the first day of drying). Biomass was machine-chopped with a particle size of approximately 2.5 cm in length and loaded into a stainless-steel tub with a 45.4L (12 gallon) capacity for distillation. Distillation was run for 30 min from start to finish with the first drop of oil emergence occurring 2–3 min into the run. The source of steam was a water heater and the steam flow manually regulated to provide a condensation temperature of 32.2–35.0 °C. Oil was collected with a 50 mL syringe and stored in 50 mL glass bottles. A picture of the distillation plant is available in Fig. S5.

### Mint oil analysis

Oil samples were analyzed on an Agilent 7890A system (Agilent Technologies, Inc., Santa Clara, CA, USA) equipped with a flame ionization detector (FID) using a 30 m PEG column (Size: 30 m, Ø = 0.25 mm, film thickness 0.25 µm) with hydrogen as the carrier gas at Callisons (Lacey, WA, USA). The flow rate was set at 1 mL min^−1^ and the oven ramp protocol was set to 60–230 °C over 17 min. Compounds were identified via a retention time library established from in-house gas chromatography/mass spectrometry data. Values represent the area percentage of the entire chromatogram (an example of GC-FID chromatograms is included in Fig. S6).

### Data analysis

Data presented here represents the mean value of all field replications including standard deviations. Statistical significance was evaluated in RStudio (Version 1.2.1335) using Tukey’s multiple comparison test (p < 0.05) and ANOVA. Representation of oil analytic data was graphed with *R/superheat*^36^. Pearson correlation coefficients among different compounds found in the oil were graphed using *R/corrplot*^37^.

## Supplementary Information

Supplementary Information 1.

Supplementary Information 2.

Supplementary Information 3.

## Data Availability

The sequences reported in this paper have been deposited in the National Center for Biotechnology Information BioProject database (BioProject ID: PRJNA784153). The plant material used in this study was obtained from a public repository (the National Clonal Germplasm Repository, USDA ARS, Corvallis, OR) following the guidelines for plant material distribution. The lines used here are available at the US National Plant Germplasm System and described with their corresponding identifiers in the materials and method section. Field research experiments were conducted following the University of California ANR Environmental Health & Safety guidelines for field operations.
