# Author Correction: Diploid mint (*M. longifolia*) can produce spearmint type oil with a high yield potential

**DOI:** 10.1038/s41598-022-05238-3

**Published:** 2022-01-13

**Authors:** Nestor Kippes, Helen Tsai, Meric Lieberman, Darrin Culp, Brian McCormack, Rob G. Wilson, Eric Dowd, Luca Comai, Isabelle M. Henry

**Affiliations:** 1 Department of Plant Biology and Genome Center, University of California at Davis, UC Davis Genome Center, Davis, CA 95616 USA; 2 ANR, Intermountain Research and Extension Center, University of California Cooperative Extension, Tulelake, CA 96134 USA; 3 Ingredient Science, Mars Wrigley, 1132 W. Blackhawk St, Chicago, IL 60642 USA

Correction to: *Scientific Reports* https://doi.org/10.1038/s41598-021-02835-6, published online 07 December 2021

The original version of this Article contained errors in the smell descriptions of the two limonene isoforms.

As a result, in the Introduction section,

“Carvone isoforms are a critical flavor component of many herbs such as caraway (*Carum carvi*) or dill (*Anethum graveolens*)^15,16^, and (−)-limonene has a citric aroma characteristic of many important fruits such as orange, lemons, or grapefruits^17^.”

now reads:

“Carvone isoforms are a critical flavor component of many herbs such as caraway (*Carum carvi*) or dill (*Anethum graveolens*)^15,16^, and (−)-limonene has a piny, turpentine-like odor, unlike the D-isomer, (+)-limonene, which has citric aroma characteristic of many important fruits such as orange, lemons, or grapefruits^17^.”

Additionally, in the Results section, the subheading ‘Characterization of oil quality and kemotypes relationships’ now reads ‘Characterization of oil quality and chemotypes relationships’. Under the same subheading,

“(−)-Limonene, a cyclic monoterpene with citric aroma, was found to be negatively correlated with cis-carvyl acetate and (−)-carvone (Fig. 6).”

now reads:

“(−)-Limonene was found to be negatively correlated with cis-carvyl acetate and (−)-carvone (Fig. 6).”

The original Article has been corrected.

