# Supplementary material for: Diploid mint (M. longifolia) can produce spearmint type oil with a high yield potential
Source: Sci Rep. 2021 Dec 7;11:23521. doi: 10.1038/s41598-021-02835-6 (PMC8651677; doi:10.1038/s41598-021-02835-6)
Supplement: Supplementary file 1 — Supplementary Information 1. [file 41598_2021_2835_MOESM1_ESM.docx]

**Diploid mint (*M. longifolia*) can produce spearmint type oil with a high yield potential**

Kippes Nestor^1^, Tsai Helen^1^, Lieberman Meric^1^, Culp Darrin^2^, McCormack Brian^3^, Wilson Rob G^2^, Dowd Eric^3^, Comai Luca^1^ and Henry Isabelle M^1*^

**SUPPLEMENTAL MATERIAL**


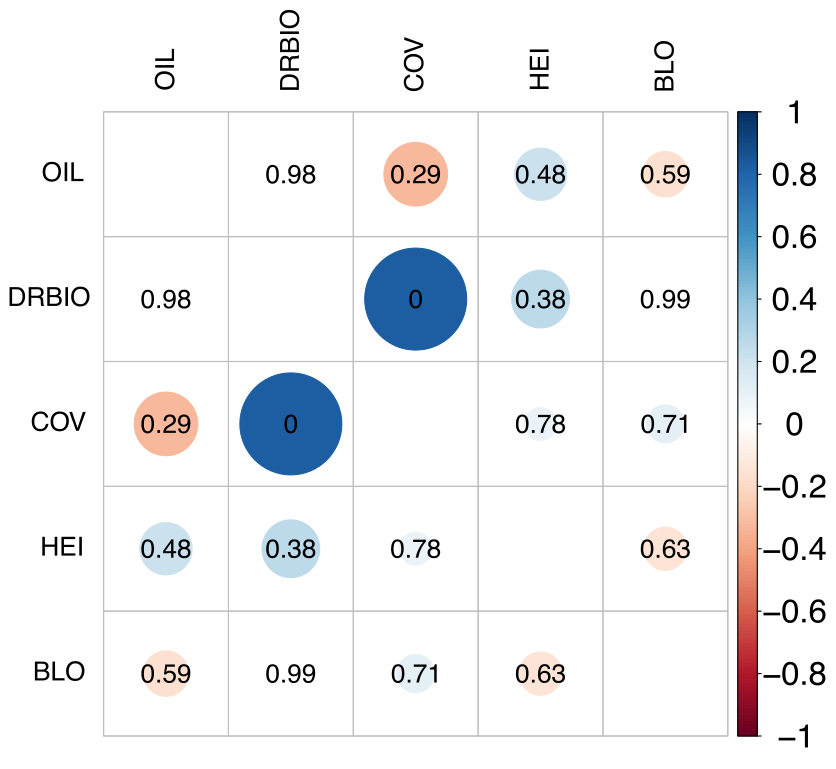


**Figure S1. Pearson's correlation coefficient for traits measured in the first field season (2018).** Plot coverage (COV) and dry biomass (DRBIO) are the only traits significatively correlated ( *p* = 0.000534). OIL: oil yield, HEI: plant height and BLO: bloom. All remaining *p* values are shown.


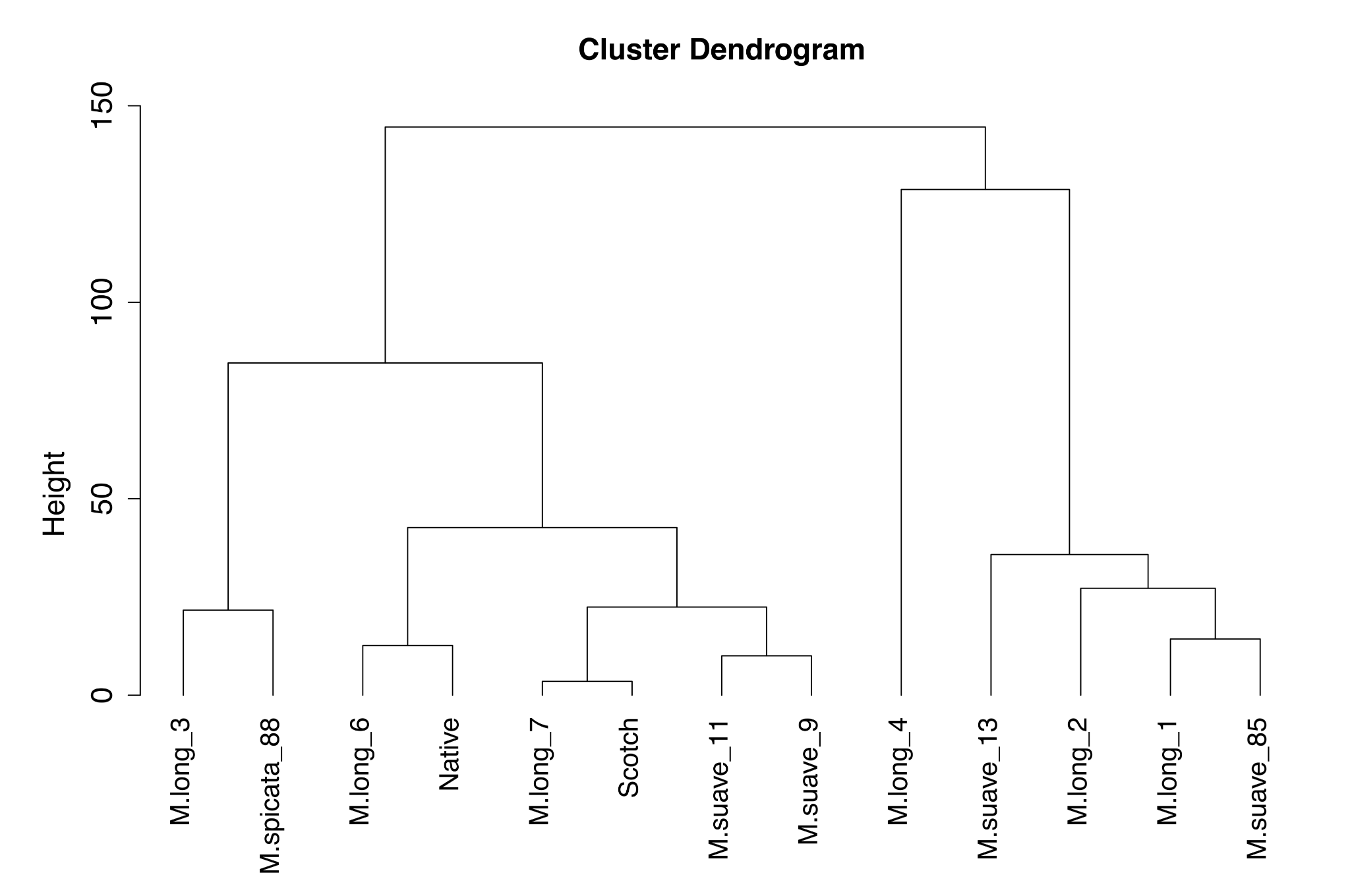


**Figure S2. Hierarchical clustering of all detected compounds found by GC-FID analysis.** M.long_7, M.suave_9 and M.suave_11 showed high levels of (-)carvone and (-)-limonene and clustered with the Scotch spearmint control (center).


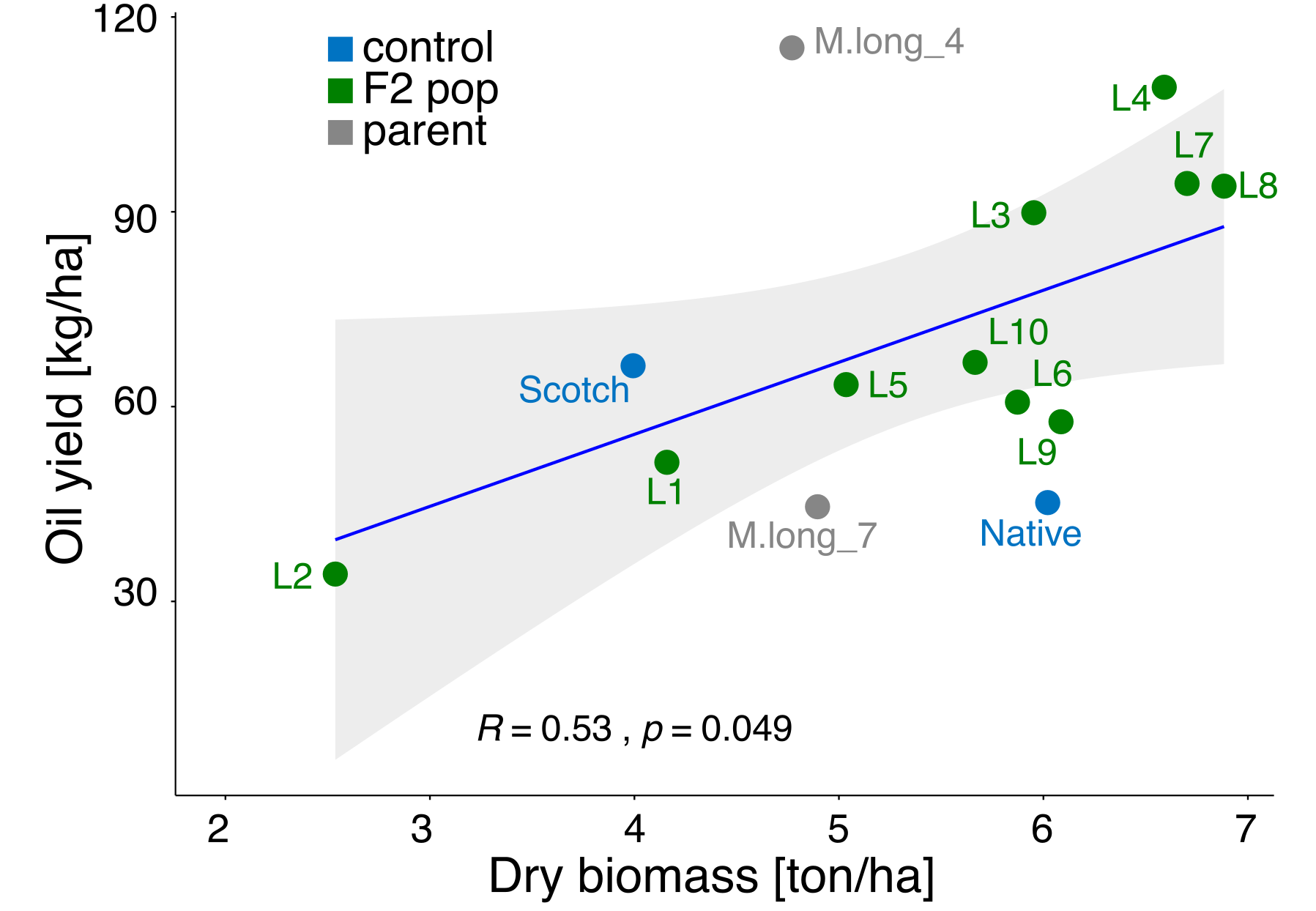


**Figure S3. Correlation between oil yield and biomass.** M.long_4 and M.long_7 are the parents of the F2 population, Native and Scotch industry controls. L1-L10 are F2 progenies. Dots represent averages of three biological replicates. We found a higher correlation when we considered only the F2 progenies (R = 0.84, *p* = 0.0026).


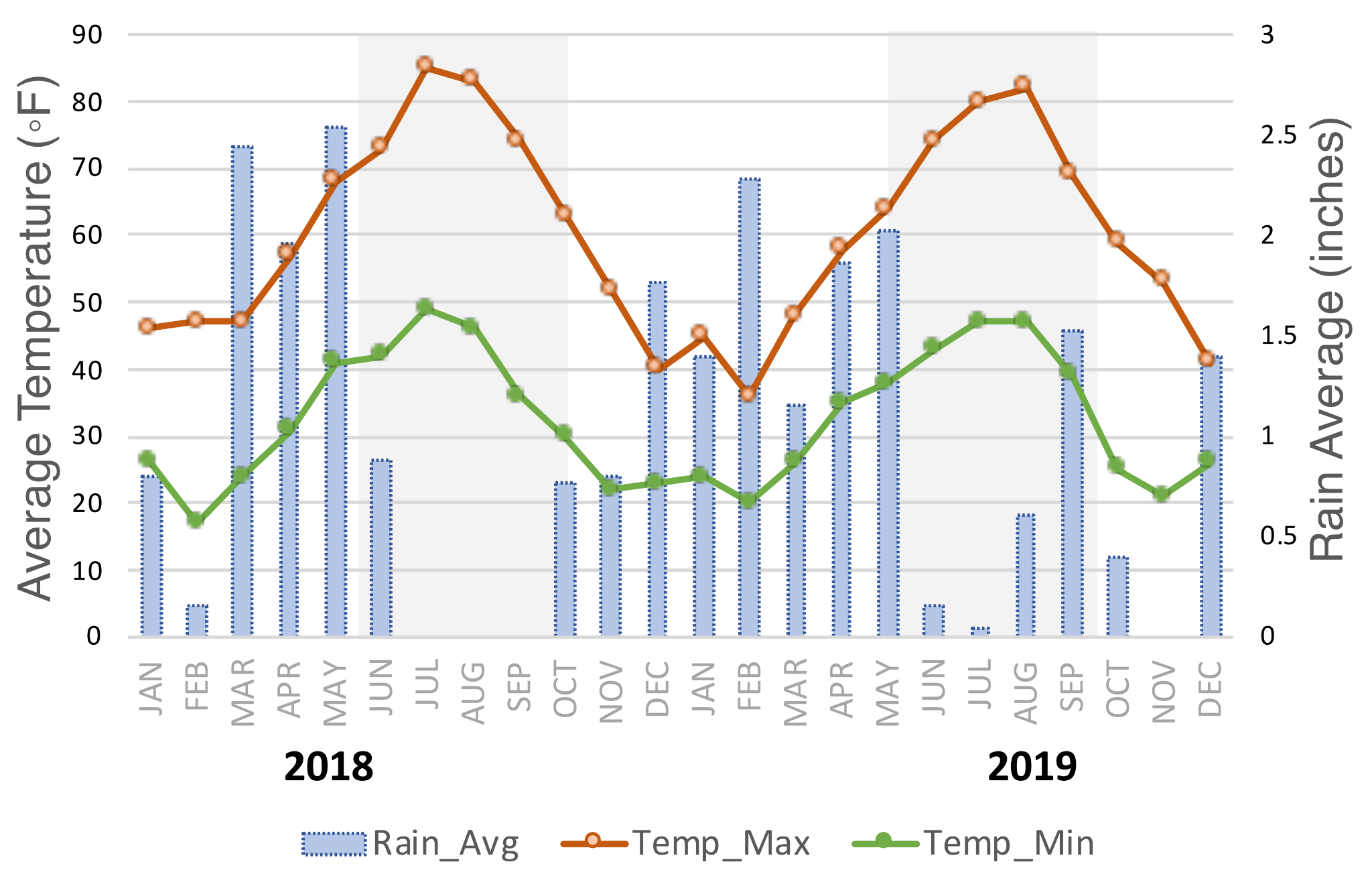


**Figure S4. Monthly average temperature (℉) and rainfall (inches) at Tulelake, California.** Data as measured by the California Irrigation Management Information System (CIMIS) weather station ([www.cimis.water.ca.gov](http://www.cimis.water.ca.gov)**)**. Growing season is highlighted with gray color.


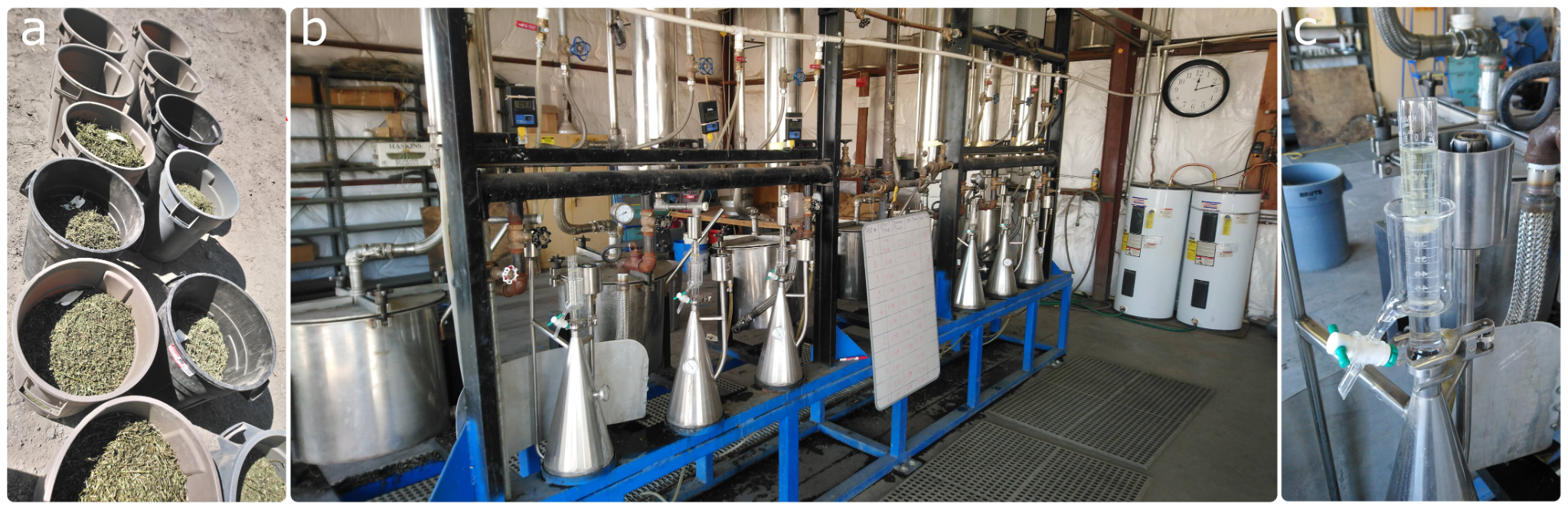


**Figure S5.** Pilot plant for steam distillation (University of California Intermountain Research and Extension Center field station at Tulelake, CA). a Picture of chopped biomass before distillation. b Overview of the pilot plant (front). c Oil during the phase separation step (oil on top, water on bottom).


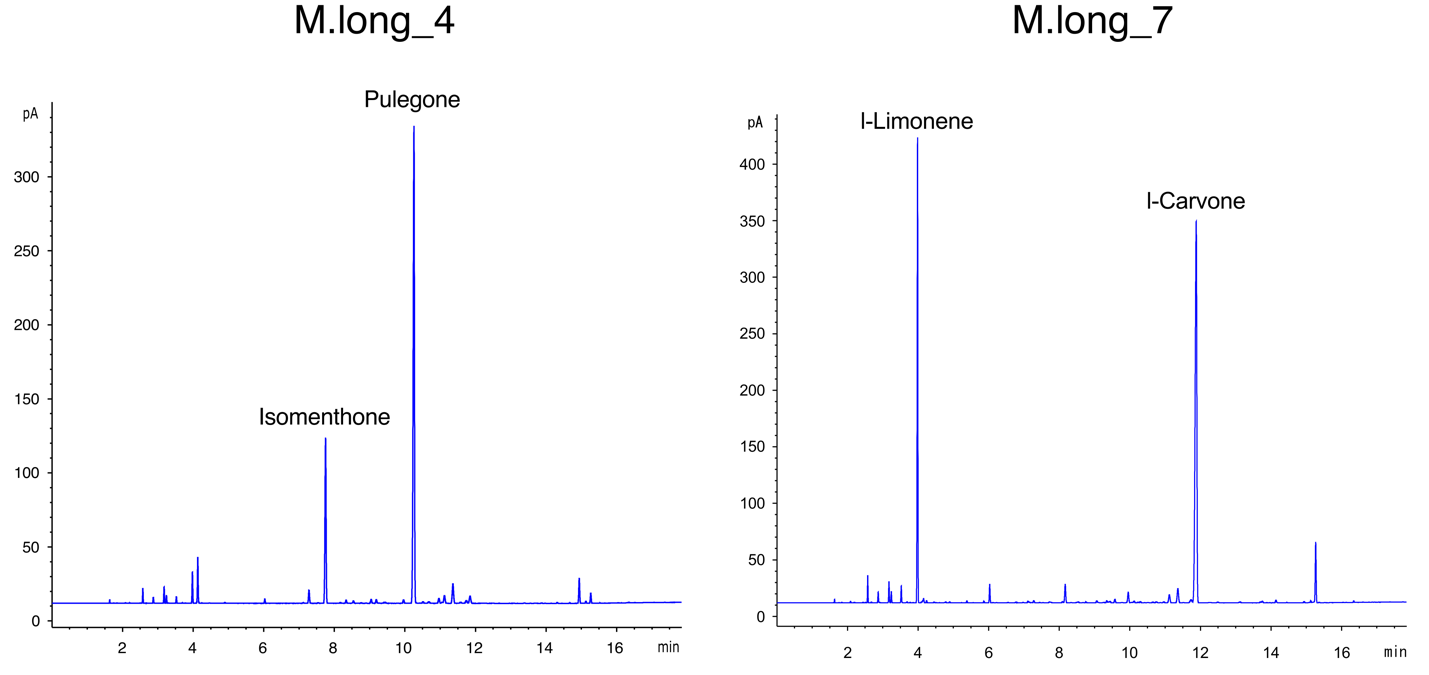


**Figure S6. GC-FID representative chromatograms.** Panels show chromatograms for the parents of the F_2_ population. The main components of the oil produced by M.long_4 are pulegone and isomenthone while, in M.long_7, the most abundant compounds are (-)-carvone and (-)-limonene.

| **This paper** | **Plant inventory** | **Plant name** | **Origin** | **Ploidy** | **Corolla color** | **Anthers** | **Growth habit** | **Leaf shape** | **Leaf tip** | **Verticillium R.** |
| --- | --- | --- | --- | --- | --- | --- | --- | --- | --- | --- |
| M.long_1 | PI 557758 | M. longifolia 10063 | India | NA | White | - | Upright | Ovate | - | S |
| 2 | PI 557770 | M. longifolia subsp. typhoides 10034 | Syria | 2x | Purple | - | Upright | Ovate | - | R |
| 3 | PI 557755 | M. longifolia 10028 | Unknown | 2x | Purple | - | Upright | Ovate | - | R |
| 4 | PI 557767 | M. longifolia subsp. capensis | South Africa | 2x | White | Yes | Upright | Lanceolate | Acute | R |
| 6 | PI 557768 | M. longifolia subsp. Hymalaiensis | Nepal | 2x | Purple | - | Upright | Ovate | - | R |
| 7 | PI 557769 | M. longifolia subsp. Polyadenia | South Africa | 2x | White | Yes | Upright | Lanceolate | Acute | S |
| M.suave_9 | PI 557638 | M. suaveolens No. 27 | France | 3x | Purple | Yes | Upright | Ovate | Obtuse | r |
| 11 | PI 557998 | M. suaveolens 10052 | Unknown | 2x | White | No | Upright | Ovate-lanceolate | Obtuse | R |
| 13 | PI 557891 | M. suaveolens 10014 2n rot. | Unknown | 3x | Purple | Yes | Upright | Ovate | Obtuse | r |
| 85 | PI 557898 | M. suaveolens 10021 | Unknown | 2x | White | Yes | Upright | Ovate | Obtuse | S |

**Table S1. Description of lines used in this study.** Plant inventory numbers correspond to USDA-GRIN database identifiers (<https://npgsweb.ars-grin.gov>). Verticillium resistance represent a qualitative estimate of the resistance level (R: resistant, S: succeptible, and r: partially resistant). Phenotypic information was obtained from previously published descriptions^1,2^ and the USDA-GRIN database (<https://npgsweb.ars-grin.gov>).

| **Genotype** | **Class** | **Oil Yield**  **[kg/ha]** | **Dry Biomass**  **[ton/ha]** | **Plot Coverage**  **[%]** | **Plant Height**  **[cm]** | **Bloom**  **[%]** |
| --- | --- | --- | --- | --- | --- | --- |
| M.long_2 | longifolia | 2.89 ± 0.88^e^ | 4.98 ± 0.17^ab^ | 97.00 ±0.71^a^ | 52.42 ± 0.44^bcde^ | 78.75 ± 5.91^abc^ |
| M.suave_13 | suaveolens | 3.17 ± 0.87^e^ | 6.13 ± 0.54^ab^ | 96.50 ± 1.50^a^ | 48.25 ± 2.19^cdefg^ | 65.00 ± 9.79^bcd^ |
| M.long_6 | longifolia | 5.70 ± 0.25^e^ | 3.47 ± 0.22^b^ | 87.25 ±3.04 ^a^ | 45.00 ± 2.72^efg^ | 95.75 ± 2.17^ab^ |
| M.suave_85 | suaveolens | 6.70 ± 0.27^e^ | 6.89 ± 1.16^a^ | 98.75 ± 0.25^a^ | 48.08 ± 1.74^defg^ | 6.50 ± 2.18^fg^ |
| M.long_1 | longifolia | 12.65 ± 1.15^de^ | 4.95 ± 0.43^ab^ | 93.75 ± 2.98^a^ | 60.00 ± 1.67^b^ | 19.50 ± 13.87^efg^ |
| M.spicata_88 | spicata | 13.60 ± 1.28^cde^ | 4.10 ± 0.50^b^ | 87.50 ± 4.33^a^ | 41.83 ± 1.00^g^ | 63.75 ± 3.75^cd^ |
| Native | control | 16.35 ± 2.01^cde^ | 4.04 ± 0.50^b^ | 88.25 ± 4.59^a^ | 43.17 ± 2.80^fg^ | 1.00 ± 0.41^g^ |
| M.long_3 | longifolia | 24.97 ± 3.23^cd^ | 6.09 ± 0.86^ab^ | 95.00 ± 3.34^a^ | 42.75 ± 2.55^fg^ | 100.00 ± 0.00^a^ |
| M.suave_11 | suaveolens | 25.59 ± 2.90^bcd^ | 6.02 ± 0.43^ab^ | 97.50 ± 0.87^a^ | 51.17 ± 0.44^bcdef^ | 78.75 ± 8.26^abc^ |
| M.suave_9 | suaveolens | 25.84 ± 1.07^bcd^ | 5.96 ± 0.52^ab^ | 97.75 ± 0.48^a^ | 57.08 ± 1.28^bc^ | 73.75 ± 4.73^abc^ |
| M.long_7 | longifolia | 27.08 ± 3.71^bc^ | 5.69 ± 0.86^ab^ | 91.25 ± 3.77^a^ | 78.42 ± 1.77^a^ | 37.50 ± 3.23^def^ |
| Scotch | control | 38.75 ± 3.55^b^ | 4.84 ± 0.57^ab^ | 93.00 ± 2.71^a^ | 44.67 ± 0.97^efg^ | 14.25 ± 6.56^efg^ |
| M.long_4 | longifolia | 73.43 ± 6.48^a^ | 4.98 ± 0.17^ab^ | 87.50 ± 2.50^a^ | 56.00 ± 1.43^bcd^ | 40.00 ± 4.08^de^ |
| **Table S2. Phenotypic data collected on season 1 (2018).** Data represent the mean of four biological replicates (plots) with corresponding standard error. Different letters indicate significantly different means (Tukey, *p* < 0.05). | | | | | | |

| **Genotype** | **Class** | **Oil Yield**  **[kg/ha]** | **Dry Biomass**  **[ton/ha]** | **Plot Coverage**  **[%]** | **Plant Height**  **[cm]** | **Bloom**  **[%]** |
| --- | --- | --- | --- | --- | --- | --- |
| L2 | F2 pop | 34.21 ± 11.78^d^ | 2.54 ± 0.98^b^ | 76.67 ± 13.33^b^ | 50.56 ± 7.87^de^ | 18.67 ± 8.95^de^ |
| M.long_7 | parent | 44.58 ± 2.95^d^ | 4.90 ± 0.28^ab^ | 100.00 ± 0.00^a^ | 84.00 ± 1.39^a^ | 36.67 ± 3.33^bcd^ |
| Native | control | 45.22 ± 4.83^d^ | 6.02 ± 1.03^a^ | 100.00 ± 0.00^a^ | 47.56 ± 3.49^e^ | 8.33 ± 3.33^e^ |
| L1 | F2 pop | 51.42 ± 4.40^cd^ | 4.16 ± 0.31^ab^ | 93.33 ± 1.67^ab^ | 70.56 ± 2.45^abc^ | 40.00 ± 0.00^bcd^ |
| L9 | F2 pop | 57.67 ± 4.64^cd^ | 6.09 ± 0.37^a^ | 98.33 ± 1.67^a^ | 86.33 ± 2.67^a^ | 50.00 ± 5.77^b^ |
| L6 | F2 pop | 60.69 ± 13.59^cd^ | 5.87 ± 0.19^a^ | 98.33 ± 1.67^a^ | 77.22 ± 1.06^abc^ | 25.00 ± 7.64^cde^ |
| L5 | F2 pop | 63.39 ± 7.50^cd^ | 5.04 ± 0.44^ab^ | 96.67 ± 3.33^ab^ | 65.56 ± 0.87^bcd^ | 40.00 ± 0.00^bcd^ |
| Scotch | control | 66.29 ± 5.09^bcd^ | 3.99 ± 2.03^a^ | 98.33 ±1.67^a^ | 51.33 ± 1.20^de^ | 76.67 ± 3.33^a^ |
| L10 | F2 pop | 66.81 ± 17.19^bcd^ | 5.67 ± 0.75^a^ | 96.67 ± 3.33^ab^ | 77.67 ± 3.01^abc^ | 40.00 ± 0.00^bcd^ |
| L3 | F2 pop | 89.86 ± 3.24^abc^ | 5.95 ± 0.17^a^ | 98.33 ± 1.67^a^ | 86.78 ± 4.82^a^ | 35.00 ± 2.89^bcd^ |
| L8 | F2 pop | 93.95 ± 2.14^abc^ | 6.88 ± 0.38^a^ | 100.00 ± 0.00^a^ | 80.89 ± 1.42^ab^ | 50.00 ± 5.77^b^ |
| L7 | F2 pop | 94.36 ± 7.55^abc^ | 6.70 ± 0.04^a^ | 100.00 ± 0.00^a^ | 80.56 ± 2.70^ab^ | 46.67 ± 3.33^bc^ |
| L4 | F2 pop | 109.18 ± 5.61^ab^ | 6.59 ± 0.05^a^ | 100.00 ± 0.00^a^ | 80.89 ± 1.44^ab^ | 43.33 ± 3.33^bc^ |
| M.long_4 | parent | 115.25 ± 12.84^a^ | 4.77 ± 0.53^ab^ | 100.00 ± 0.00^a^ | 64.17 ± 2.50^cde^ | 55.00 ± 5.00^ab^ |
| **Table S3. Phenotypic data collected on season 2 (2019).** Data represent the mean of three biological replicates (plots) with corresponding standard error. Different letters indicate significantly different means (Tukey, *p* < 0.05). | | | | | | |

**Table S4:** Supplementary materials, TableS4_2018_GCFID.xlsx

**Table S5:** Supplementary materials, TableS5_2019_GCFID.csv

| **Name** | **Sequence (5'-3')** | **Product length (bp)** | **Scaffold** |
| --- | --- | --- | --- |
| NK-001.Contig97695_F1 | TTG AGA TGC TGA GTG TGA GCT T | 829 | scaffold6 |
| NK-002.Contig97695_R1 | CCA TTG AGG TTG TTG GTG TG |  |  |
| NK-005.Contig7547_F1 | AGA GCT GCG AGA CAA AGG TA | 1207 | scaffold4 |
| NK-006.Contig7547_R1 | AGA ACT CCA AAA GCA TGC GG |  |  |

**Table S6.** Primer sequences of the PCR markers used to confirm F1 hybrids obtained from crosses between the *M. longifolia* accessions.

**REFERENCES**

1. [Vining, K. J., Zhang, Q., Tucker, A. O., Smith, C. & Davis, T. M. Mentha longifolia (L.) L.: A model species for mint genetic research. *HortScience* **40,** 1225–1229 (2005).](http://paperpile.com/b/VOGoNj/LWUZ)
2. [Vining, K. J., Pandelova, I., Hummer, K., Bassil, N., Contreras, R., Neill, K., Chen, H., Parrish, A. N. & Lange, B. M. Genetic diversity survey of Mentha aquatica L. and Mentha suaveolens Ehrh., mint crop ancestors. *Genet. Resour. Crop Evol.* **66,** 825–845 (2019).](http://paperpile.com/b/VOGoNj/WXja)
